# Activated Carbon from Tropical Almond Shell (*Terminalia catappa L.*): Synthesis, Characterization, Application, and Regeneration for Chromium (VI) Adsorption

**DOI:** 10.1021/acsomega.6c06329

**Published:** 2026-10-22

**Authors:** Tiago Magnago, José Jailton Marques, Victor Hugo dos SantosRamos, Danilo Conceição Silva, Licianne Pimentel Santa Rosa, Edilson de Jesus Santos

**Affiliations:** † Postgraduate Program in Chemical Engineering, 74391 Federal University of Sergipe, Avenida Marechal Rondon, s/n, Rosa Elze, São Cristóvão 49100-000, Sergipe, Brazil; ‡ Postgraduate Program in Environmental Engineering and Sciences, Federal University of Sergipe, Avenida Marechal Rondon, s/n, Rosa Elze, São Cristóvão 49100-000, Sergipe, Brazil; § School of Chemical Engineering, Postgraduate Program in Chemical Engineering (PPGEQ), 28132 University of Campinas, Campinas 13083-852, São Paulo, Brazil; ∥ Department of Chemical Engineering, Federal University of Sergipe, Avenida Marechal Rondon, s/n, Rosa Elze, São Cristóvão 49100-000, Sergipe, Brazil

## Abstract

This study reports the synthesis of activated carbon (AC–H_3_PO_4_) from tropical almond shell (*Terminalia catappa L.*), an agro-waste material, using phosphoric acid and evaluates its effectiveness in removing hexavalent chromium (Cr­(VI)) from aqueous media, emphasizing regeneration and reuse as strategies aligned with circular economy principles. The adsorbent was characterized in terms of its textural properties and chemical composition using Brunauer–Emmett–Teller (BET) surface area analysis and scanning electron microscopy, zero charge point (PCZ), EDX, and elemental analysis (CHN). Batch adsorption studies were performed to evaluate the removal of Cr­(VI) from aqueous solutions. Textural analysis showed that the material CA-H_3_PO_4_ has a high mesoporous area (54%), with surface area BET of 1489.72 m^2^ g^–1^. The pH at which charges were neutralized between the adsorbent surface and the solution was equivalent to 2. At this pH, Cr­(VI) removal exceeded 80.0% under the experimental conditions of 100 mL of solution containing 50.0 mg L^–1^ Cr­(VI) and 0.05 g of the adsorbent. The adsorption kinetics were evaluated using the pseudo-first-order, pseudo-second-order, and Weber–Morris intraparticle diffusion models, with the pseudo-second-order model providing the best fit. Likewise, the equilibrium data were analyzed using the Langmuir, Freundlich, and Temkin isotherm models, with the Freundlich model showing the best agreement with the experimental data. The adsorption process was rapid, exhibiting a rate constant of 0.002 g mg^–1^ min^–1^, and was found to be spontaneous and endothermic, as indicated by the values of Δ*G* (−0.2 to −0.5 kJ mol^–1^) and Δ*H* (13.3 kJ mol^–1^). Desorption experiments demonstrated that the adsorbent could be regenerated using 0.5 mol L^–1^ NaOH, retaining a Cr­(VI) adsorption capacity of 83.6 mg g^–1^ after the fourth regeneration cycle, corresponding to 55.0% of its original adsorption capacity. These findings suggest that CA-H_3_PO_4_ has considerable potential as an adsorbent for the remediation of waters contaminated with hexavalent chromium.

## Graphical abstract

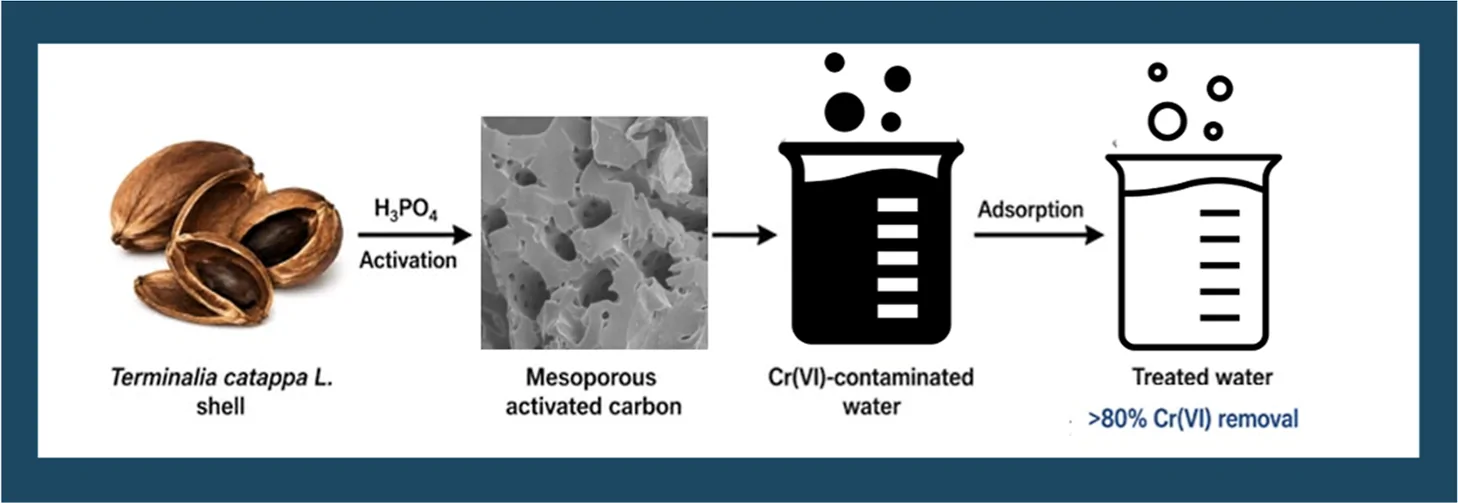

## 1 Introduction

Industrial development has triggered the abuse of available resources, as well as the unregulated disposal of industrial waste in the environment. Any metal element with relatively high density compared to water and considered toxic even at low concentrations is called a heavy metal.^1^ Heavy metal pollution has become a global issue as concentrations above allowable levels become an imminent danger to any living organism. Most heavy metals such as arsenic (As), cadmium (Cd), lead (Pb), mercury (Hg), and chromium (Cr) are known for their toxicity, nonbiodegradability, persistence in nature, and tendency to bioaccumulate.^2^ Among these, chromium, which occurs predominantly in trivalent and hexavalent forms, has a range of applications in the stainless-steel industry, electroplating, dyeing, leather tanning, and wood treatment processes.^1,3^

According to the World Health Organization (WHO), the maximum permitted limit for Cr­(VI) in drinking water is 0.05 mg L^–1^.^4^ Therefore, it is important to remove this contaminant from wastewater prior to its emission to the recipient bodies. There are several methods developed for the treatment of Cr­(VI) contaminated waters, including chemical precipitation, ion exchange, membrane separation processes, electrodialysis, photocatalytic degradation, and adsorption.^5^ Adsorption has emerged as an attractive treatment option because of its high removal efficiency, even at low concentrations, combined with simple operation and relatively low costs.

Activated carbon (CA) is a carbonaceous adsorbent obtained from the pyrolysis of high-porosity lignocellulosic materials, capable of selectively adsorbing gases, liquids, metal ions, or other contaminants inside their pores.^6^ Costs related to the adsorption technology can be reduced by using a low-added-value precursor in the production of the adsorbent. Therefore, several researchers have been intensifying the use of agricultural residues, biomass, and other solid residues in the manufacture of activated carbon. Recent research points to CA production from Paulownia wood,^7^ walnut shells,^8^ apple shell,^3,9^ coconut shells,^10^ peanut shells and wood from Jatropha,^11^ and shells of *Prosopis juliflora*,^12^ babassu coconut,^13^ coconut coir fiber,^14^ moringa,^15^ almond pomace,^16^ etc. A mesoporous biochar with a high specific surface area (986.35 m^2^ g^–1^) was reported to achieve more than 99% removal of malachite green dye under optimized adsorption conditions.^17^ An activated biochar derived from water lily stems also exhibited over 99% removal of the same dye within 30 min at room temperature through a combination of physisorption and chemisorption mechanisms.^18^ Likewise, raw *Ipomoea batatas* leaves and the corresponding biochar were successfully applied for the adsorption of Procion Orange MX-2R dye, demonstrating the promising performance of both adsorbents and their potential for textile wastewater treatment.^19^ Vamvuka et al.^20^ investigated modified almond kernel biochar for ammonium removal and found that KOH and dolomite modification improved the adsorption capacity, reaching up to 40.6 mg g^–1^, highlighting its potential for wastewater treatment. Pertile et al.^21^ compared almond shell-derived biochars produced by slow and microwave pyrolysis for Pb­(II) removal and found that nonactivated biochar obtained by slow pyrolysis showed the best adsorption performance, highlighting the potential of this low-cost and sustainable material for water treatment. Wang et al.^22^ developed ZnCl_2_-modified almond shell biochar for dye removal, achieving a combined adsorption capacity of 696 mg g^–1^ for crystal violet and methylene blue, demonstrating high efficiency and good reusability for water treatment. Barzallo et al.^23^ synthesized a magnetic biochar@ZIF-8 composite from almond shell for fluoroquinolone removal, exhibiting high adsorption performance and maintaining over 84% of its capacity after four reuse cycles, highlighting its potential as a sustainable and magnetically recoverable adsorbent for water treatment.

Carbonaceous materials produced from thermal processing (pyrolysis or carbonization) are commonly activated to increase specific surface area as well as pore fraction. Activation methods are divided into physical and chemical ones. In chemical activation, a carbonaceous precursor is doped with a chemical agent and porosity is developed by dehydration and oxidation reactions.^24^ The most commonly used activating agents found in the literature are H_3_PO_4_,^3,7,11,25^ NaOH,^26^ ZnCl_2_,^8,27,28^ KOH,^11,29^ and H_2_SO_4_.^9,12^ Phosphoric acid (H_3_PO_4_) is widely used because it promotes dehydration reactions, increases carbon yield, and develops well-defined micro- and mesoporous structures with high specific surface area at relatively low activation temperatures.^29^ However, its application requires extensive washing to remove residual phosphate species, generating acidic wastewater and increasing water consumption and post-treatment requirements.^30^

Among the biomasses used to produce activated carbon for Cr­(VI) removal, considerable research efforts have been devoted to the production CA from almond shells (*Prunus dulcis*). Rai et al.,^31^ for example, prepared microporous activated carbon from almond shell powder and activated with H_3_PO_4_ and was used for the removal of Cr (VI). The Cr (VI) removal was found to be 100% at an initial pH of 2. Yüksel and Orhan^32^ used activated carbon produced from a mixture of fruit stones for Cr­(VI) removal in a fixed-bed column. The results showed a decrease in removal performance with increasing flow rate, pH, and temperature, while an improvement was observed with greater bed depth and higher influent metal ion concentration. In the study by Shu et al.,^33^ almond shell derived activated carbon modified with nano zerovalent iron was applied for Cr­(VI) removal, achieving nearly 90% removal efficiency within 60 min. Kali et al.,^34^ investigated the ability of lignocellulosic waste materials derived from almond shells to remove hexavalent chromium from the aquatic environment. Guo et al.^35^ proposed a novel almond shell activated carbon modified with FeS and chitosan. They investigated the adsorption capacity of heavy metals such as cadmium (Cd), lead (Pb), and chromium (Cr (VI) and Cr (III)) from water and soil. The authors observed that activated carbon modified with FeS is an efficient adsorbent for the removal of heavy metals. Adsorption efficiencies exceeding 90% were reported. Altun et al.^36^ synthesized activated carbon from almond shells via pyrolysis at 400 and 500 °C and developed composite adsorbents by coating them with chitosan for Cr­(VI) removal from aqueous solutions. These investigations have mainly emphasized the synthesis, characterization, and adsorption performance of such materials.

Although biomass-derived activated carbons have been extensively investigated for Cr­(VI) removal, studies have predominantly focused on their preparation, characterization, and initial adsorption performance, whereas their regeneration and reuse have remained comparatively less explored. This limitation is particularly relevant for the practical application of adsorbents as repeated regeneration and reuse can reduce material consumption, waste generation, and treatment costs. Therefore, the present study focuses on the production of activated carbon from *Terminalia catappa* shells as a strategy for biomass waste valorization, with particular emphasis on its Cr­(VI) adsorption performance, regeneration, and reuse, thereby assessing its potential within a circular economy framework.

In this context, this study aims to synthesize activated carbon from tropical almond shells (*T. catappa L.*), an agroresidue, by chemical activation with phosphoric acid and to evaluate its performance for the removal of hexavalent chromium (Cr­(VI)) from aqueous solutions, within a circular economy framework that promotes waste valorization and resource recovery. Regeneration and reuse cycles were also investigated to assess the stability and practical applicability of the developed materials. Therefore, this work addresses an existing gap in the literature by simultaneously examining both the adsorption performance and the regeneration behavior of activated carbon derived from tropical almond shells, reinforcing their potential as a sustainable and circular alternative for water treatment applications. Therefore, the adsorbent was characterized by BET surface area analysis, scanning electron microscopy (SEM), EDX, CHN elemental analysis, and determination of the point of zero charge. Its performance for Cr­(VI) removal from aqueous solutions was evaluated in the batch mode, and the adsorption behavior was interpreted by using kinetic, isotherm, and thermodynamic models. Regeneration studies were conducted to assess the reusability of the materials. On the basis of these considerations and in line with waste minimization and reuse strategies, the fruit of the tropical almond tree (*T. catappa L.*) was selected for the purposes of this study, aiming to add value to a material with limited known applications while contributing to the mitigation of real environmental problems.

## 2 Materials and Methods

### 2.1 Materials

Phosphoric acid (85%, P.A., Dynamics), potassium dichromate (P.A., Dynamics), acetone (P.A., Neon), hydrochloric acid (37%, P.A., Neon), sulfuric acid (P.A., Neon), sodium hydroxide (P.A., Neon), and 1,5-diphenylcarbazide (P.A., Vetec) were used in this study. Distilled water (7 μS cm^–1^) was used for solution preparation and adsorbent washing.

### 2.2 Raw Material for Adsorbent Production

The material used in the production of activated carbon (CA) was the fruit of the tropical almond tree (*T. catappa L.*). The samples were collected at the campus of the Federal University of Sergipe (UFS), located in the city of São Cristóvão—SE, Brazil. Initially, the fresh raw material underwent a cleaning procedure with running water to remove impurities adhered to the shell. Then, the material was dried in a solar greenhouse, where it remained until the epicarp dehydration (outside), avoiding the proliferation of fungi. The next step was the removal of the dehydrated peel and the opening of the fruit to remove the almond (seed), thus obtaining the material of interest (shell), which was milled (TECNAL, model TE-633), classified granulometrically and reserved. The fraction of particles with diameter in the range of 1.4 mm to 4.75 mm was used in the synthesis of the adsorbent.

### 2.3 Activated Carbon Preparation

The preparation of the activated carbon was based on the method reported by Yorgun and Yıldız.^7^ Impregnation of the material was performed with H_3_PO_4_ (85%; *d* = 1.69 g mL^–1^) as an activating agent. The ratio used was 4:1, expressed as the mass ratio between H_3_PO_4_ and the precursor material. For the impregnation, 28.64 g of biomass was added to 80 mL of phosphoric acid, packed in an open becker, and the final solution volume was distilled with water up to 430 mL. The mixture was heated to approximately 85 °C for 6 h to ensure access of the activating agent into the particles. After treatment, the impregnated solid was separated from the solution by vacuum filtration and dried at 105 °C for 12 h to remove excess acid.

Carbonization and activation of the impregnated material were carried out simultaneously in a tubular reactor under an inert atmosphere, injecting nitrogen gas at a flow of 1 L min^–1^. System temperature was raised to 25 °C min^–1^ and when it reached 400 °C, it remained so for 1 h. After natural cooling, the material was washed successively several times with distilled water at 80 °C and then with distilled water at room temperature, aiming to remove the ash formed during the pyrolysis process. At the end of the washing process, the material was oven-dried at 105 °C for 24 h, thus obtaining the activated carbon (CA-H_3_PO_4_), which was graded granulometrically and stored for later use in the adsorption tests. The yield of CA-H_3_PO_4_ produced was calculated by the ratio between the final adsorbent mass obtained and the initial mass of fresh biomass, according to eq 1.

1 $${\%}_{Yield}=\left(\frac{m_{activatedcarbon}}{m_{precursor}}\right)x100$$

### 2.4 Characterization of CA-H_3_PO_4_

The textural properties were analyzed by the 77K nitrogen physisorption technique, using a Quantachrome NOVA model 1200e physical adsorption analyzer. The sample was degassed under vacuum at 200 °C for 2 h before measurement. Surface area (*S*_BET_) was determined by the method proposed by Brunauer, Emmett, and Teller (BET). The Dubinin–Radushkevich (DR) method was used to calculate the microporous surface area (Smicro). The mesoporous surface area (Smeso) was calculated by the difference between *S*_BET_ and Smicro (Smeso = SBET—Smicro). Total pore volume (VT) was estimated to be the volume of pores occupied under high relative pressure (*P*/*P*0 ∼ 0.99). Micropore volume (Vmicro) was obtained by the DR method, and mesopore volume (Vmeso) was calculated by subtracting Vmicro from VT (Vmeso = VT—Vmicro). The pore size distribution was determined by the method proposed by Barrett, Joyner, and Halenda (BJH).^37^

SEM analysis was performed to study surface texture and porosity development using a Carry Scope model JCM-5700. The chemical composition was analyzed using the following techniques: energy-dispersive X-ray fluorescence—EDX (using a Shimadzu model EDX-720HS) and elemental analysis—CHN (using a LECO elemental analyzer model CHN628).

The zero charge point (PCZ) was determined by the method known as the 11 point experiment.^33^ 0.05 g of CA-H_3_PO_4_ was measured and added to 50 mL of aqueous solution under 11 different initial pH conditions: 2, 3, 4, 5, 6, 7, 8, 9, 10, 11, and 12. The mixture was stirred at 25 °C for 24 h. Then, the final pH of each solution was measured in order to find the zero charge point of the adsorbent obtained by the difference between the initial and final pH.

### 2.5 Adsorption Experiments

Adsorption batch assays were based on the study by Yang et al.^25^ Adsorption assays were based on the study by (K_2_Cr_2_O_7_) to 1000 mg L^–1^, dissolving 2.83 g of the solute in distilled water and making up to 1 L. Working solutions were obtained by diluting the stock solution, which was placed in containers containing predefined amounts of adsorbent and stirred for pre-established intervals. Equilibrium Cr­(VI) ion concentrations were determined by the diphenylcarbazide method,^25^ from the absorbance read at 540 nm using a Biospectro spectrophotometer, model SP-220, with the amount adsorbed per mass of adsorbent at equilibrium *q*_*e*_ (mg g^–1^), calculated by eq 2

2 $$q_{e}=\left[\frac{\left(C_{0}-C_{E}\right)}{m}\right]\times V$$

where *C*_0_ and *C*_E_ (mg L^–1^) are the concentration of Cr­(VI) initial and final (equilibrium) in solution, respectively, *m* is the adsorbent mass used (g), and *V* is the volume of the solution (L).

In order to find the ideal pH that favored the adsorption process, the adsorbent material was tested at different pH (2, 4, 6, 8, and 10). The tests were performed by adding 0.05 g of carbon to 100 mL of solution containing 50% Cr­(VI) ions mg L^–1^. The adsorbent material remained in contact with the solution for 2 h under stirring at 150 rpm at room temperature. The pH was adjusted with sodium hydroxide (NaOH) and hydrochloric acid (HCl), both at 1 mol L^–1^, according to the pH of interest. The percentage of removal (*R*_*e*_) was calculated by eq 3

3 $$R_{e}=\left[\frac{\left(C_{0}-C_{E}\right)}{C_{0}}\right]\times 100$$

where *C*_0_ and *C*_E_ are the initial and equilibrium concentrations, respectively.

### 2.6 Adsorption Kinetics

For the kinetic study, 0.05 g of de CA-H_3_PO_4_ was added to 100 mL of K_2_Cr_2_O_7_ (50 mg L^–1^) and subjected to stirring at 150 rpm under a constant temperature of 25 °C. Samples were collected and analyzed at different time intervals in the range of 5 to 360 min. Adsorption kinetics were evaluated using the pseudo-first and pseudo-second order. The PSO model equally relates several vacant sites available for the adsorption on adsorbent, they assume that adsorption rate is controlled by the chemical process (chemisorption).^38^

The adsorption mechanism was investigated using the Weber–Morris intraparticle diffusion model, following the methodology proposed by Kumar and Kumaran.^39^ Intraparticle diffusion was used to determine type of the adsorption mechanism in the process.^18^ ^,^ ^40^

### 2.7 Adsorption Isotherms

In order to investigate Cr­(VI) adsorption capacity in CA-H_3_PO_4_, adsorption isotherms were obtained using the following procedure: 0.05 g of the adsorbent was added to 100 mL of K_2_Cr_2_O_7_ in different initial concentrations (25, 40, 50, 75, 100, 150, 200, and 250 mg L^–1^) and subjected to agitation at 150 rpm under constant temperature of 25 °C. The samples were collected and analyzed after the equilibrium time, determined in item 2.6. Adsorption equilibrium was further evaluated using two isotherm models.^34^ ^,^ ^41^

### 2.8 Adsorption Process Thermodynamics

The thermodynamic parameters for the adsorption process were obtained by the procedure described in item 2.7, at different temperatures (15, 25, and 35 °C), calculated from the system of equations presented below, according to a study by Huang et al.^42^

4 $$\Delta G=-RT\ln\left(K_{C}\right)$$

where *R* is the universal gas constant (8.314 J mol^–1^ K^–1^), *K*_C_ is the equilibrium constant, and T­(K) is the temperature. Standard enthalpy (Δ*H*) and adsorption entropy (Δ*S*) can be estimated from the van’t Hoff eq (eq 5).

5 $$\ln\left(K_{C}\right)=-\frac{\Delta H}{RT}+\frac{\Delta S}{R}$$

The slope and intercept of the graph of $\frac{1}{T}$ versus ln­(*K*_C_) are qual to $-\frac{\Delta H}{R}$ and $\frac{\Delta S}{R}$ , respectively.

### 2.9 Adsorbent Regeneration

CA-H_3_PO_4_ was first saturated with the adsorbate, leaving 0.05 g of the adsorbent in 100 mL of 150 mg L^–1^ solution containing Cr (VI) under constant stirring (at 150 rpm) for 6 h at 25 °C. Desorption was performed by transferring the saturated carbon to a beaker containing a 0.5 mol L^–1^ NaOH solution. For each 0.15 g of the saturated adsorbent, 100 mL of leach solution was used and the system was stirred for 12 h. Washing with distilled water was then followed by immersion in 0.5 mol L^–1^ HCl solution for 2 h, followed by filtration, washing with distilled water and drying at 105 °C to find CA-H_3_PO_4_ regenerated.^4^ All adsorption experiments were performed in triplicate, calculating the standard deviation at each point.

## 3 Results and Discussion

### 3.1 Characterization of CA-H_3_PO_4_

Production yield, defined as mass of activated carbon per unit of raw biomass mass, was 40.5%. The result was quite similar to that found by Niazi et al.,^26^ who reported 54% yield; A. Kumar & Jena,^8^ approximately 31% and Yorgun and Yıldız,^7^ com 30%, used phosphoric acid as an activating agent and under the following process conditions: temperature, 450, 600, and 400 °C; impregnation ratio, 1:1, 2:1, and 4:1; carbonization and activation time, 150, 60, and 60 min, respectively. The raw material, virgin activated carbon (CA-H_3_PO_4_), and Cr­(VI) saturated activated carbon were analyzed for their elemental composition. The result of this characterization is shown in Table 1.

**Table 1:** Composition of Fresh Raw Material and Virgin and Saturated Adsorbent—Organic Fraction^a^

elements	M.P *in natura* [mass (%)]	CA-H_3_PO_4_ [mass (%)]	CA-H_3_PO_4__Cr(IV) [mass (%)]
C	89.29	57.83	55.93
H	2.76	4.31	4.99
N	0.48	0.31	0.29
O^a^	7.47	26.01	23.57

a Obtained by difference.

High carbon feedstocks are preferred for activated carbon production.^35^ In this context, the almond tree (*T. catappa L.*) is an appropriate precursor for the production of this adsorbent as it is made up of 89.29% carbon. After pyrolysis and activation, there is an increase in the amount of oxygen and a decrease in carbon, this fact is associated with the activating agent. The activation process with H_3_PO_4_ promotes a dehydration reaction, resulting in the formation of different phosphate and polyphosphate groups, increasing the structure oxygen content and permanent carbon loss.^43^ Sorption of Cr­(VI) changes the composition only for inorganic components.

Table 2 shows the inorganic composition of virgin activated carbon and after the adsorption process. It should be noted that the most representative percentage was P_2_O_5_ from the chemical activation process. Other components except chromium are present in the composition of lignocellulosic biomass,^43^ which even after the carbonization and activation steps remained in the activated carbon structure. The fraction of chrome present in [CA-H_3_PO_4__Cr­(VI)] is the result of the adsorption process.

**Table 2:** Composition of CA-H_3_PO_4_ before and after the Adsorption—Inorganic Fraction^a^

components	CA-H_3_PO_4_ [mass(%)]	CA-H_3_PO_4__Cr(VI) [mass(%)]
K_2_O	0.01	0.03
P_2_O_5_	10.60	6.29
SO_3_	0.17	0.14
Fe_2_O_3_	0.02	0.02
SiO_2_	0.74	0.27
Cr_2_O_3_	N/D^a^	8.47

a Not detected.

The textural characteristics of the adsorbent such as surface area (*S*_BET_), total pore volume (*V*_T_), microporous characteristics (S_micro_ and *V*_micro_), mesoporous characteristics (S_meso_ and *V*_meso_), and mean pore diameter (*D*_p_) are presented in Table 3. The material obtained has a well-developed surface area and total pore volume, 1489.72 m^2^ g^–1^ and 0.823 cm^3^ g^–1^, respectively. The data show that 53.7% of the total quantified area consists of mesopores.

**Table 3:** Results of CA-H_3_PO_4_ Textural Characterization

*S*_BET_ (m^2^ g^–1^)	*S*_micro_ (m^2^ g^–1^)	*S*_meso_ (m^2^ g^–1^)	*V*_T_ (cm^3^ g^–1^)	*V*_micro_ (cm^3^ g^–1^)	*V*_meso_ (cm^3^ g^–1^)	*D*_p_ (nm)
1489.72	689.73	799.99	0.823	0.379	0.444	2.849

Figure 1 shows the pore distribution, which shows that the adsorbent material consists mainly of micropores (<2 nm) and mesoporous (2 to 50 nm), according to IUPAC, however, the mesoporous volume occupies 53.7% of the total. This micro- and mesoporous predominance was already expected since activation with phosphoric acid allows the development of pores exclusively in this range.^37^

**Figure 1:**
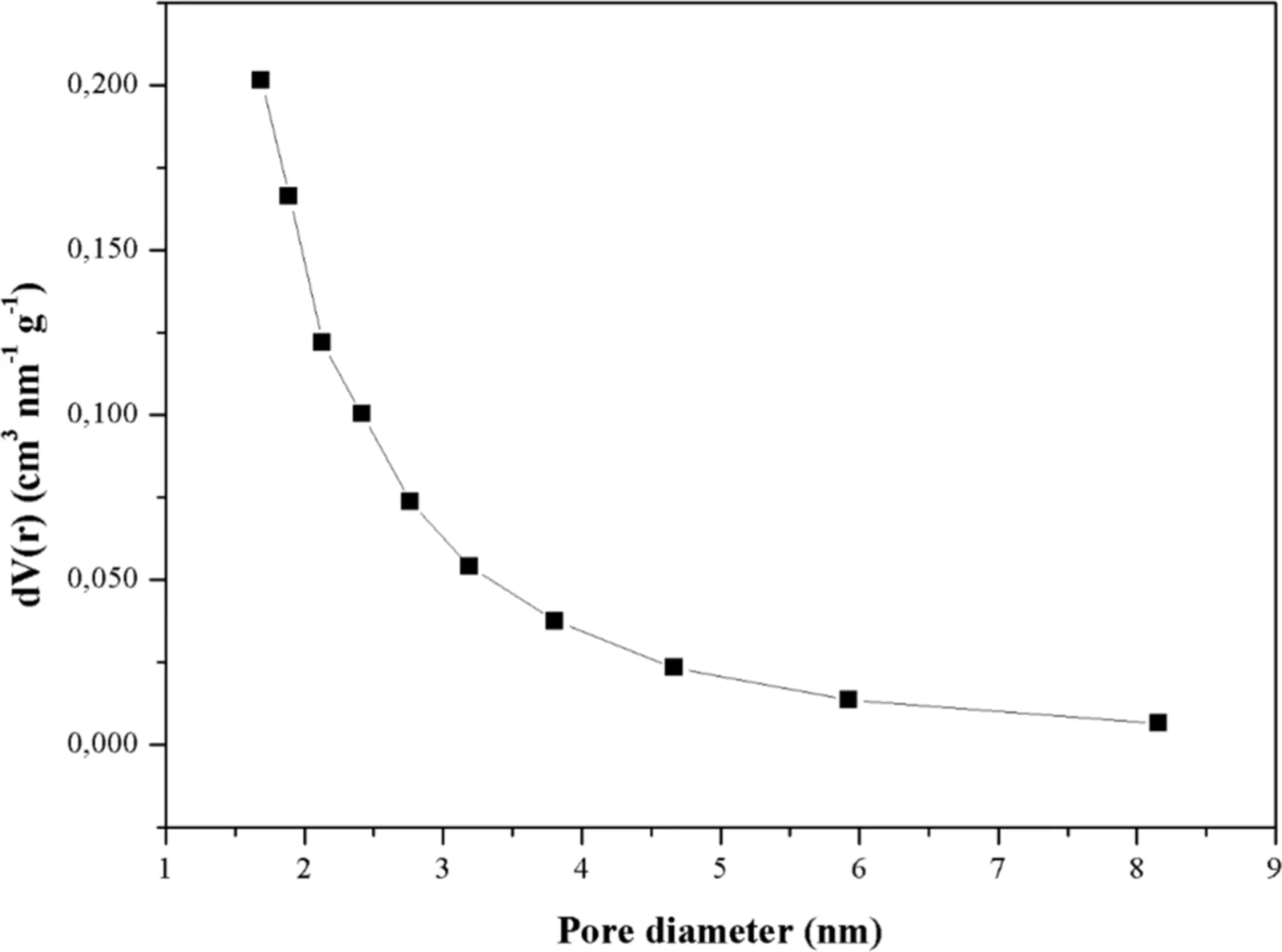
CA-H_3_PO_4_ pore distribution.

Figure 2 shows the morphological analysis of the biochar (nonactivated carbon) (Figure 2a) and the activated carbon (CA-H_3_PO_4_) (Figure 2b), characterized by SEM. The micrographs reveal homogeneous, irregular, and porous surfaces for both of the materials.

**Figure 2:**
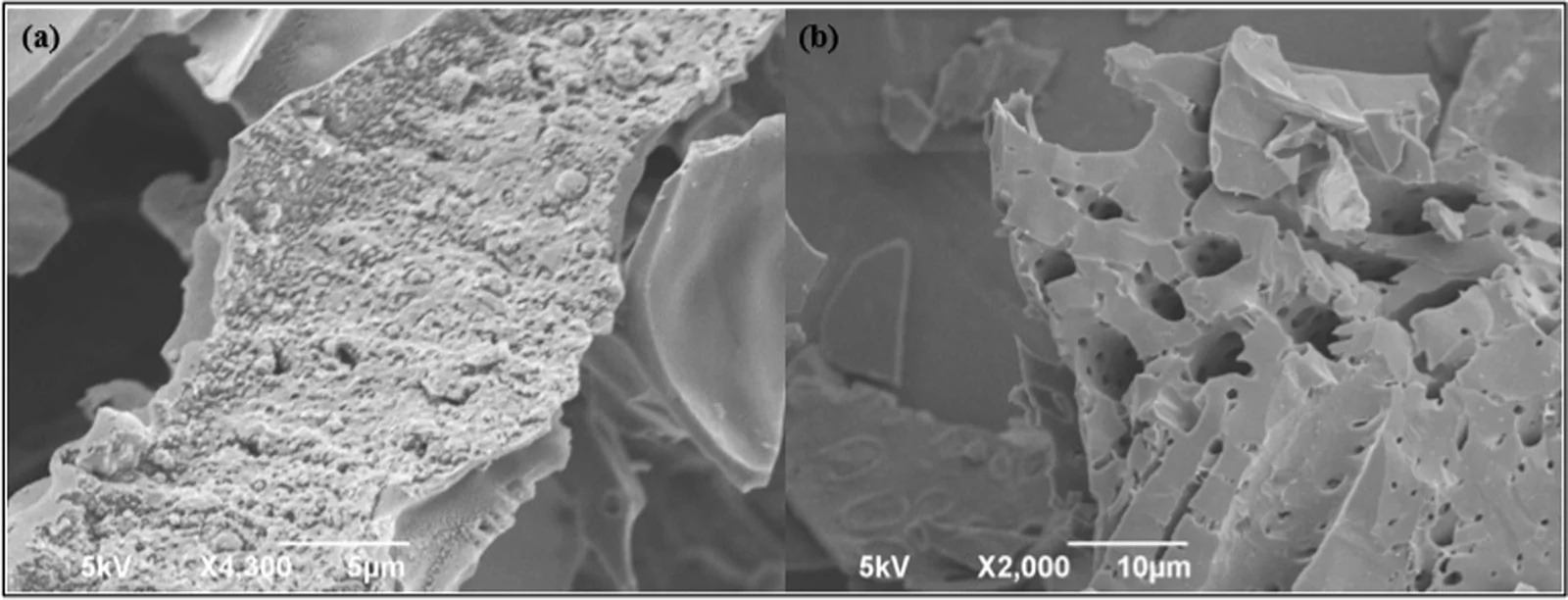
Micrographs of samples (a) carbon without activation and (b) CA-H_3_PO_4_.

SEM images of the raw biomass and activated carbon are presented in Figure 2. The images were acquired at different magnifications to better illustrate the most representative morphological features of each material. The raw biomass required higher magnification to reveal its scarce surface pores, whereas the activated carbon exhibited a highly developed porous network that was readily observed at a lower magnification. Despite the different magnifications, the images clearly demonstrate the significant morphological changes induced by the chemical activation process including the development of a more porous and heterogeneous surface. The biochar micrograph (Figure 2a) indicates that the material has a predominant but undeveloped porous structure with partially clogged pores. The activated carbon SEM image (Figure 2b) shows a more developed, clean, and unobstructed porosity, so it can be inferred that its pore network is larger, with a cylindrical shape, enlarged by the activating agent. In addition, it is possible to observe the presence of smaller pores inside, which make the adsorbent highly porous.

The results obtained in the test for the determination of the zero-charge pH are shown in Figure 3. It is observed that the pH at which charges were neutralized between the surface of the adsorbent and the solution was 2, which detracted from ion exchange between the surface of the adsorbent and the aqueous medium. The same behavior is observed at a pH close to 12. Another characteristic of CA-H_3_PO_4_ is its protonated surface in all studied pH ranges, favoring the adsorption of negative molecules.

**Figure 3:**
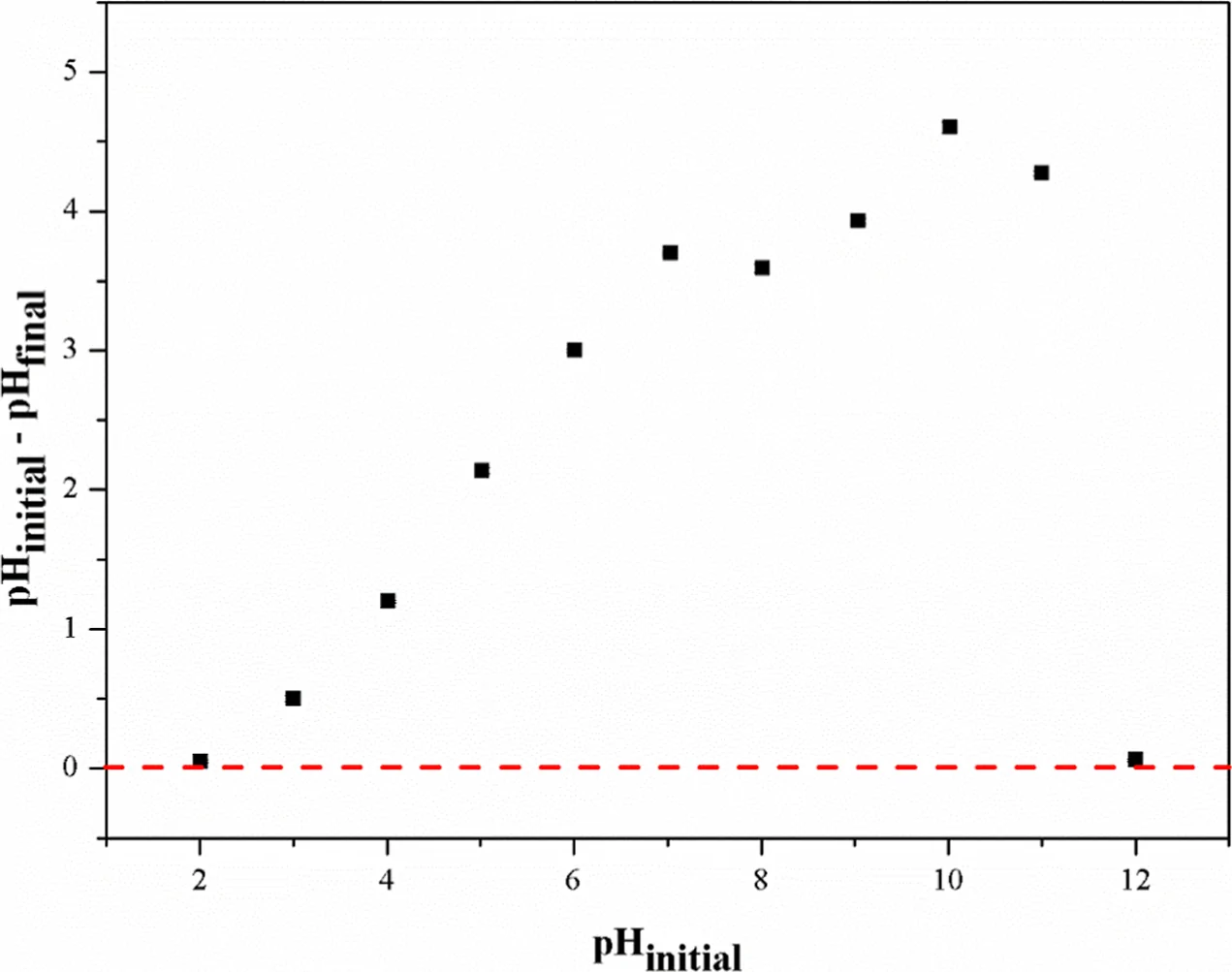
CA-H_3_PO_4_ zero charge pH.

### 3.2 Adsorption Experiments

#### 3.2.1 Effect of pH on Cr­(VI) Removal

The pH of the aqueous solution is an important parameter for the control of the heavy-metal adsorption process.^4^ The influence of pH was first evaluated at a Cr­(VI) concentration of 50 mg L^–1^ and a dosage of 0.05 g activated carbon at different initial pH values, as shown in Figure 4.

**Figure 4:**
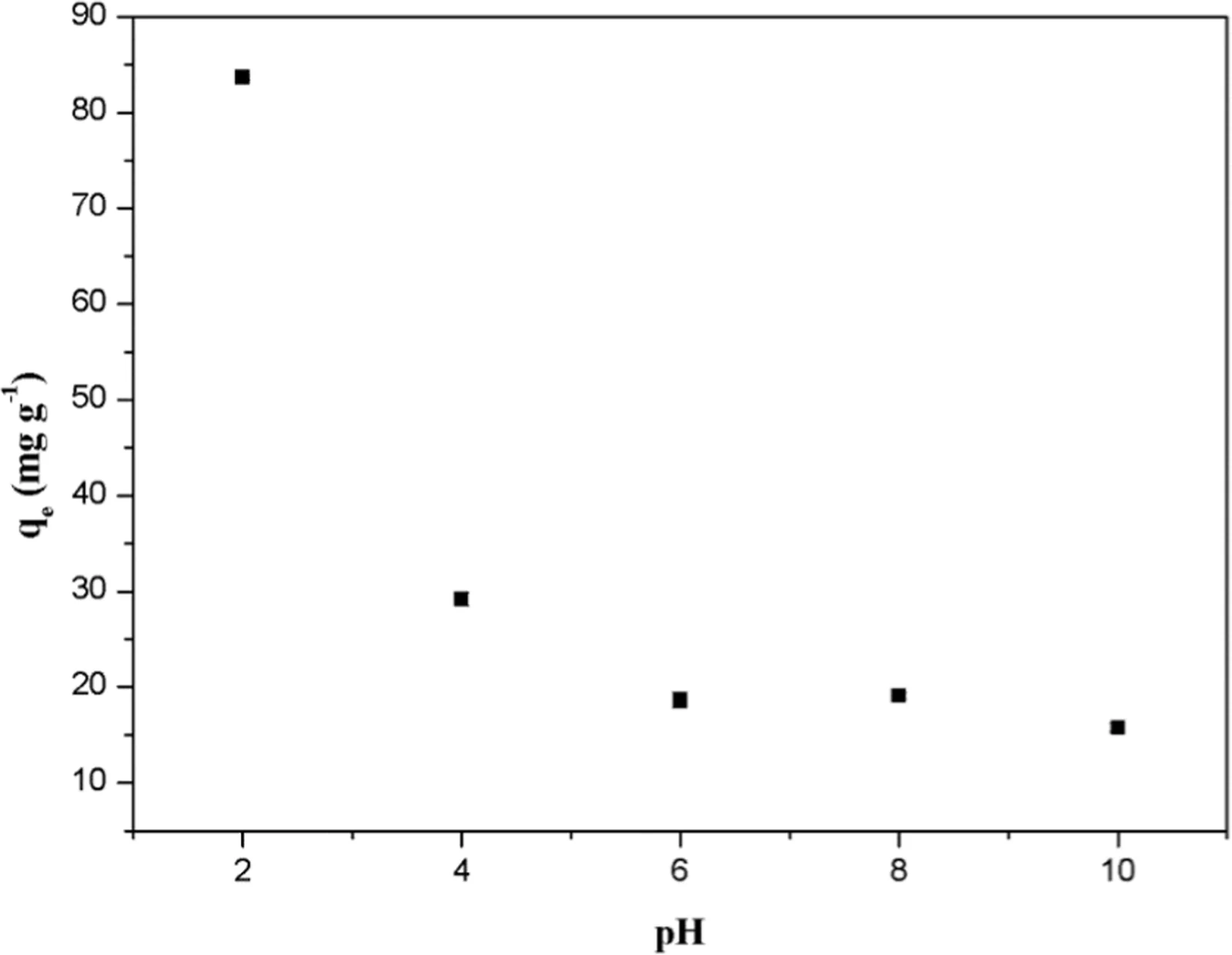
Effect of pH on Cr­(VI) adsorption in CA-H_3_PO_4_.

The Cr­(VI) adsorption capacity is higher at pH 2 (83.7 mg g^–1^) and decreases with increasing pH. The reason for this pH-dependent adsorption is due to the surface chemical composition of the adsorbent and the species of dissolved Cr­(VI)-containing ions, which change with the pH of the medium. Based on the distribution diagram, the Cr­(VI) chemical species in solution are CrO_4_^–2^, Cr_2_O_7_^–2^, H_2_CrO_4_, and HCrO_4_^–^. In an acidic environment, anionic species (such as HCrO_4_^–^) are dominant and therefore can be adsorbed on the positive surface of the adsorbent by electrostatic attraction. As the pH increases (pH > 3), the protonated surface of the adsorbent is weakened, and the dominant Cr­(VI) species are converted to CrO_4_^–2^. Therefore, the percentage of removal is reduced because of the repulsion between the chrome anions and the surface of CA-H_3_PO_4_. Another reason for the decrease in adsorption is correlated with CrO_4_^–2^ because the species occupies two free active sites of the carbonaceous structure, while HCrO_4_^–^ only one.^4^ ^,^ ^26^ Thus, all adsorption assays were performed at a pH of 2.

#### 3.2.2 Adsorption Kinetics

The effect of contact time on Cr­(VI) adsorption by CA-H_3_PO_4_ at pH 2 was investigated, and the results are shown in Figure 5. The adsorption rate is rapid in the early stage, reaching a capacity of 70.5 mg g^–1^ in 1 h. By prolonging the contact time, adsorption rates become slower, so that equilibrium is reached within 5 h. In order to study the Cr­(VI) adsorption kinetics in CA-H_3_PO_4_, the commonly applied pseudo-first-order and pseudo-second-order kinetic models were used to adjust the kinetic data.

**Figure 5:**
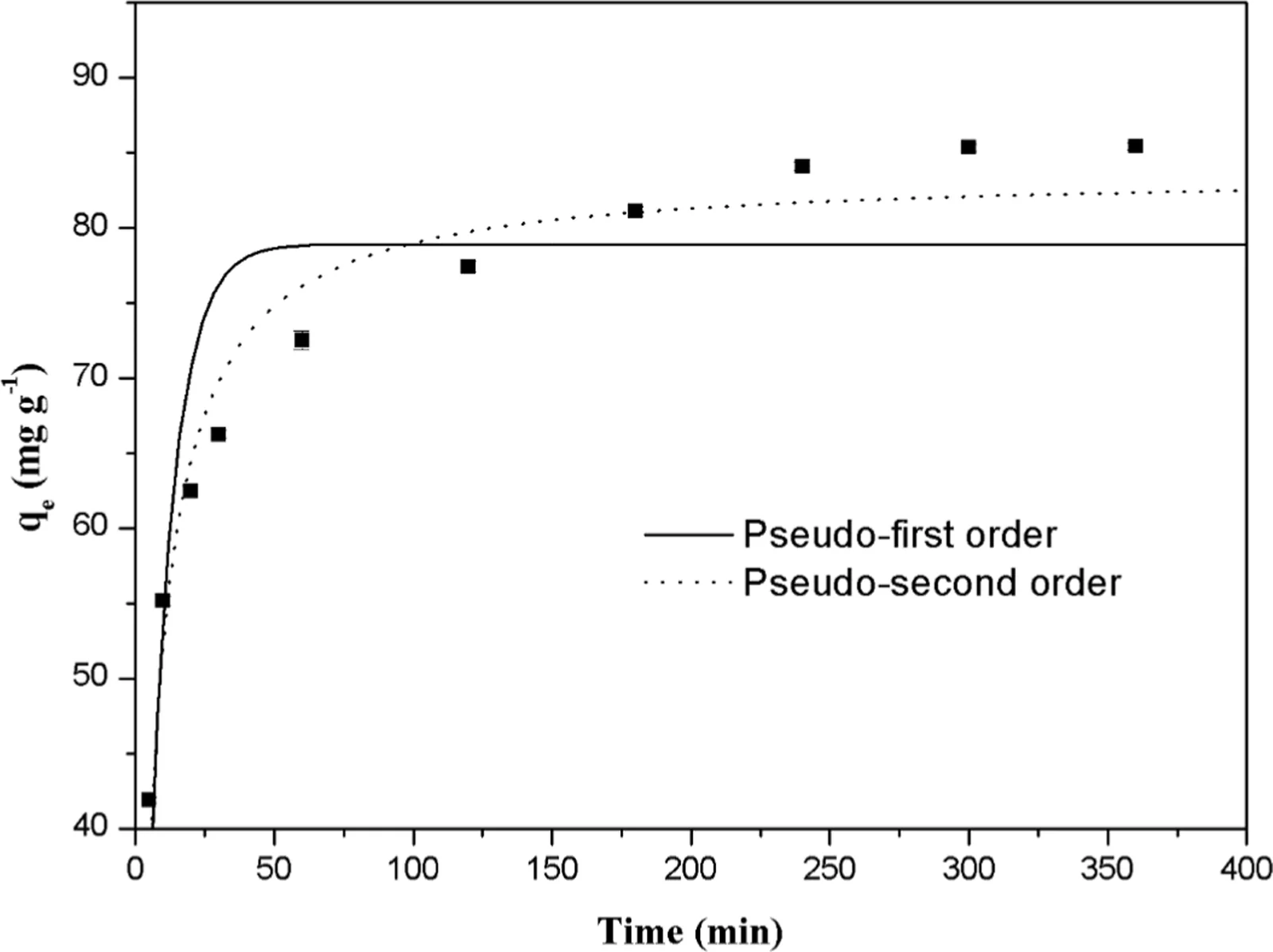
Adsorption kinetic curve of Cr (VI) on CA-H_3_PO_4_.

The values of constants and rates were calculated using the General Algebraic Modeling System (GAMS) version 145 × 86/MS, whose results are presented in Table 4. The pseudo-second-order model best described the experimental data (*R* ^2^ = 0.980), confirmed by the chi-square (χ^2^) statistical test with nine degrees of freedom and 95% confidence level, with a theoretical value of,^44^ against the obtained value of 1.319. In addition, the value of *q*_e.cal_ (83.7 mg g^–1^), calculated from the pseudo-second-order equation is similar to that obtained experimentally (*q*_e.exp_ = 85.3 mg g^–1^), indicating that the second-order model is suitable for the observed kinetics.

**Table 4:** Kinetic Parameters for Cr (VI) Adsorption in CA-H_3_PO_4_

*q*_e.exp_ (mg g^–1^) 85.3	pseudo-first-order model				pseudo-second-order model			
	*q*_e.cal_ (mg g^–1^)	*k*_1_ (min^–1^)	*R* ^2^	χ^2^	*q*_e.cal_ (mg g^–1^)	*k*_2_ (g mg^–1^min^–1^)	*R* ^2^	χ^2^
	78.9	0.113	0.903	6.133	83.7	0.002	0.980	1.319

Additionally, the intraparticle diffusion mechanism was evaluated using the Weber–Morris model, as presented in Figure 6.

**Figure 6:**
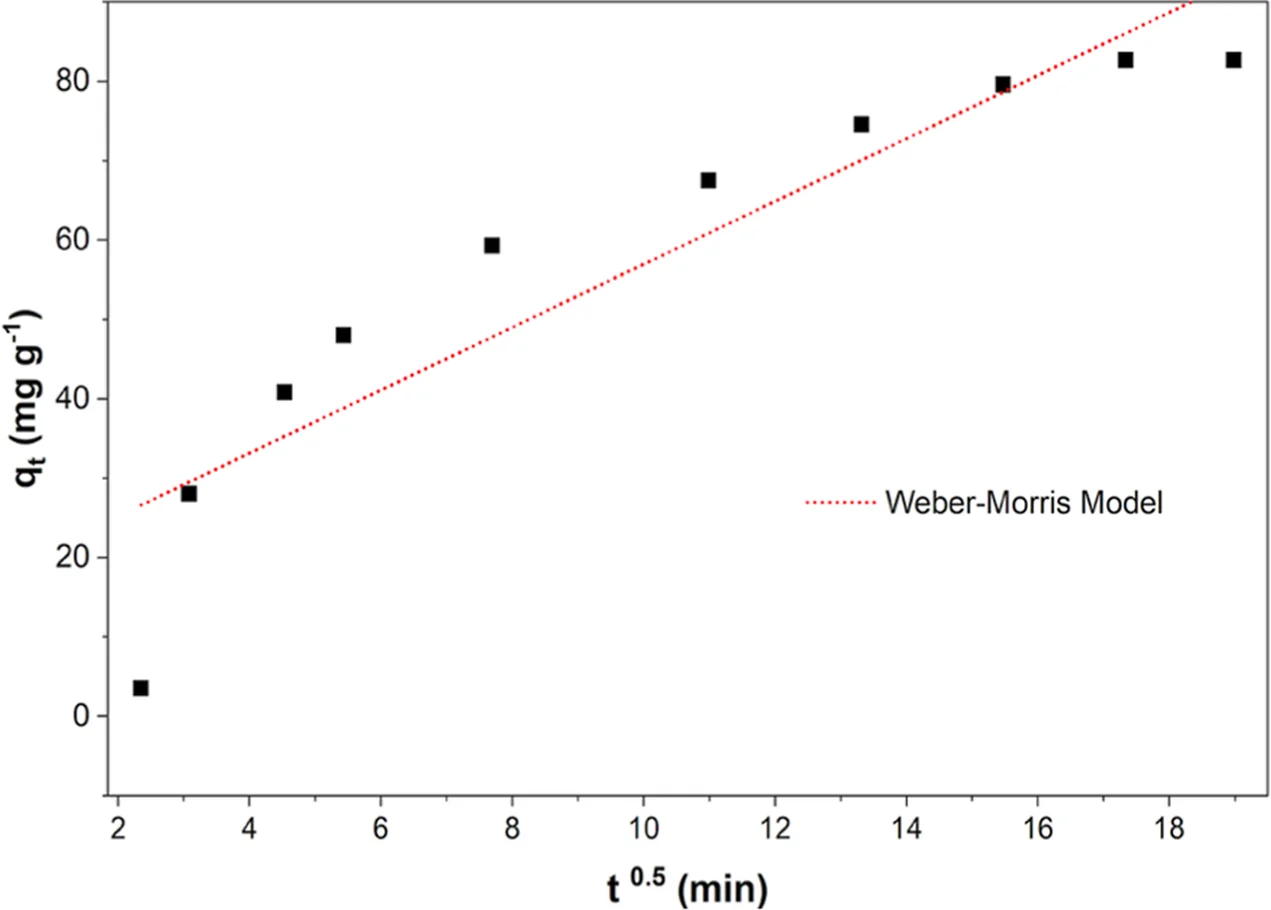
Weber–Morris intraparticle diffusion plot for Cr­(VI) adsorption onto CA-H_3_PO_4_.

The kinetic data were fitted to the model, yielding an intraparticle diffusion rate constant *k*_id_ (g mg^–1^ min^–1/5^) of 4.3704, an intercept (C) of 14.809, and a coefficient of determination (*R* ^2^) of 0.8545. The relatively high value of C and the fact that the fitted line did not pass through the origin indicate that intraparticle diffusion was not the sole rate-controlling step, suggesting that boundary layer diffusion also contributed to the adsorption process.^45^

#### 3.2.3 Adsorption Isotherms

In order to investigate the capacity and thermodynamics of the Cr (VI) adsorption process in CA-H_3_PO_4_, isotherms were obtained at three different temperatures (15, 25, and 35 °C), as illustrated in Figure 7 for the Langmuir and Freundlich isotherms and in Figure 8 for the Temkin isotherm.

**Figure 7:**
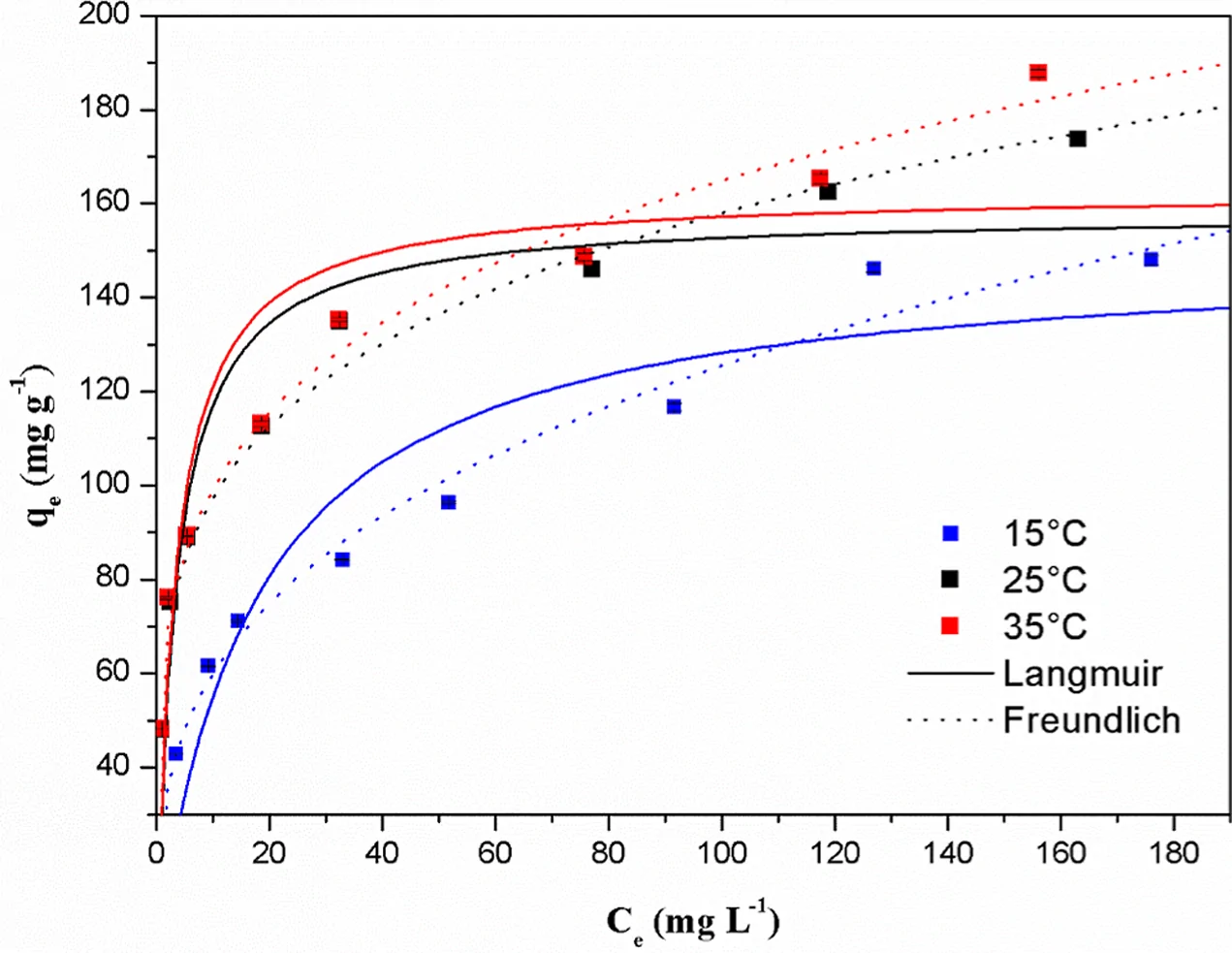
Langmuir and Freundlich adsorption isotherms for Cr­(VI) adsorption onto CA-H_3_PO_4_.

**Figure 8:**
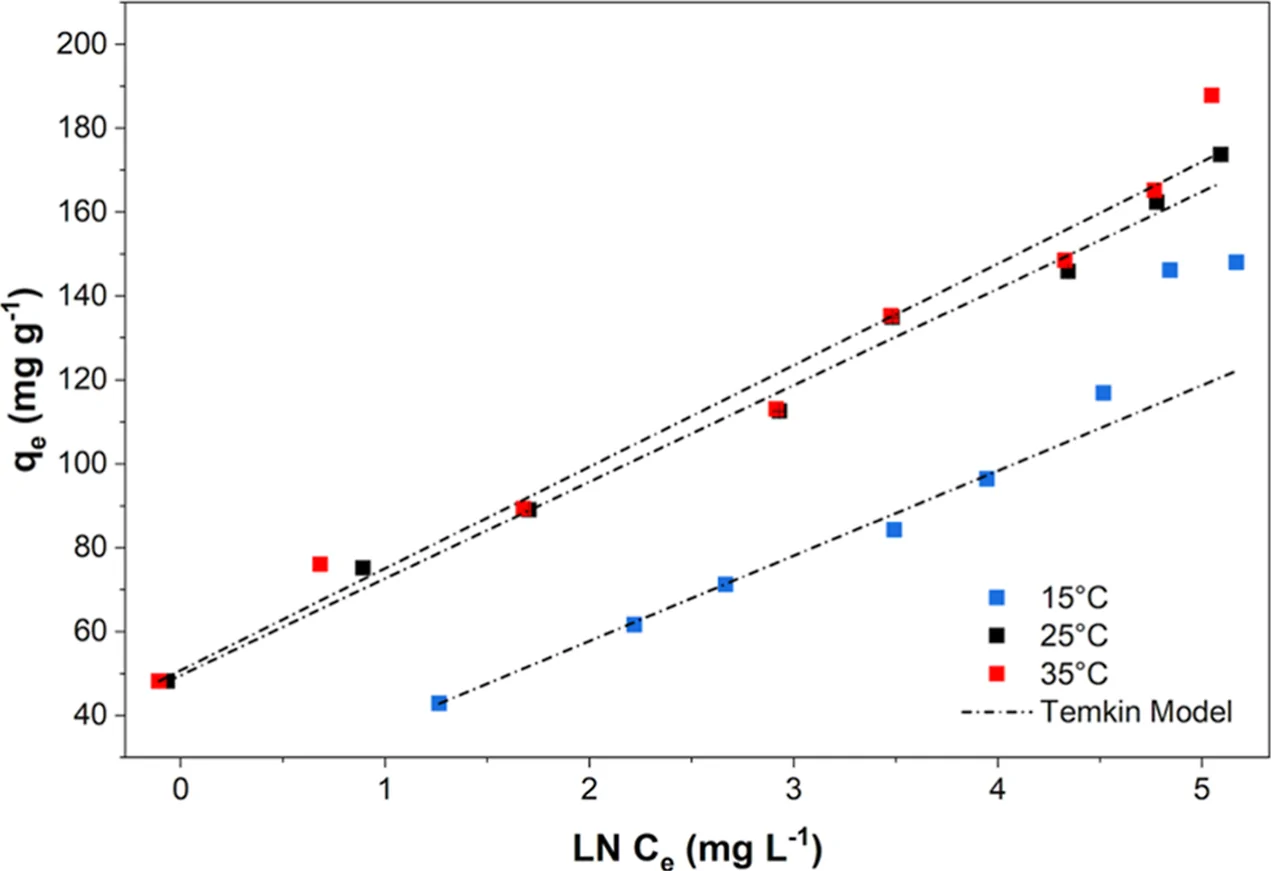
Temkin adsorption isotherm for Cr­(VI) adsorption onto CA-H_3_PO_4_.

Figure 7 shows that isotherms are “favorable” type,^43^ showing high affinity between activated carbon and Cr­(VI) ions even at low concentrations, due to the large number of accessible sites, followed by plateau, representing the maximum saturation capacity in the monolayer, which can best be seen in the curve corresponding to the 15 °C. For the other temperatures, the system did not saturate in the concentration range studied. However, it can be stated that the maximum adsorption capacities were as follows: 148.0 mg g^–1^ (15 °C), 173.7 mg g^–1^ (25 °C), and 187.8 mg g^–1^ (35 °C). Moreover, it is noted that the adsorption process was favored by the temperature increase, which can be attributed as a positive advantage to the adsorbent.

To establish the most appropriate correlation for equilibrium curves and to estimate isotherm parameters, the Langmuir and Freundlich models were adjusted to the experimental data. Table 5 presents the isotherm parameters and the quality of the adjustment for Cr­(VI) adsorption in CA-H_3_PO_4_, at all temperatures studied.

**Table 5:** Langmuir and Freundlich Isothermal Parameters for Cr­(VI) Adsorption in CA-H_3_PO_4_

temperature (°C)	Langmuir model					Freundlich model			
	*q*_max_ (mg g^–1^)	*K*_L_ (L mg^–1^)	*R*_L_	*R* ^2^	χ^2^	*K*_f_	*n*	*R* ^2^	χ^2^
15	150.3	0.058	0.065	0.946	20.955	28.679	3.119	0.990	1.906
25	158.0	0.287	0.014	0.955	15.188	60.102	4.754	0.991	1.906
35	162.5	0.294	0.013	0.933	22.420	59.700	4.533	0.990	2.007

For the Freundlich model at the three temperatures studied, the minimum Pearson correlation coefficient *R* ^2^ value was 0.99, and the chi-square statistical test with seven degrees of freedom and 95% confidence level indicated a satisfactory fit between experimental and predicted data. The theoretical theory would be 2.167,^44^ demonstrating that this model presented the best fit to the experimental equilibrium data, describing a nonideal and reversible adsorption, not restricted to the monolayer formation.^46^ *N* values in the range of 2 to 10 represent good affinity between the adsorbent and adsorbate; 1 to 2 imply moderate affinity, and values less than 1 imply low affinity.^27^ Thus, the adsorption process of Cr­(VI) by CA-H_3_PO_4_ is favorable over the whole temperature range evaluated.

The Temkin model was also applied to evaluate the adsorption equilibrium (Figure 8), considering adsorbent–adsorbate interactions and the linear decrease in the heat of adsorption with increasing surface coverage. The obtained fitting parameters are presented in Table 6.

**Table 6:** Temkin Isothermal Parameters for Cr­(VI) Adsorption in CA-H_3_PO_4_

temperature (°C)	Temkin model			
	B (mg L^–1^)	*A*_L_ (L mg^–1^)	*b*_T_ (J mol)	*R* ^2^
15	27.205	1.0129	88.0603	0.9406
25	23.234	8.3368	103.1109	0.9906
35	24.201	8.6804	98.9909	0.9694

The Temkin isotherm model was applied to further investigate the adsorption equilibrium behavior of Cr­(VI) onto CA-H_3_PO_4_, considering the adsorbent–adsorbate interactions and the gradual decrease in adsorption heat with increasing surface coverage.^46^ As presented in Table 6, the Temkin model provided satisfactory correlation coefficients (*R* ^2^ = 0.9406–0.9906), indicating that this model can adequately describe the adsorption process. The highest fitting accuracy was observed at 25 °C (*R* ^2^ = 0.9906), suggesting a more pronounced contribution of adsorbate–adsorbent interactions under this condition.

The Temkin parameter *b*_T_ (J mol), related to the adsorption heat and surface interactions, ranged from 23.234 to 27.205 mg L^–1^, showing relatively small variations with temperature. The calculated *b*_T_ (J mol) values were in the range of 88.06–103.11 J mol^–1^, indicating the presence of favorable interactions between Cr­(VI) species and the functional groups present on the CA-H_3_PO_4_ surface. The Temkin equilibrium constant *A*_L_ (L mg^–1^) increased considerably from 1.0129 L mg^–1^ at 15 °C to approximately 8.5 L mg^–1^ at higher temperatures, suggesting an enhancement of the interaction between Cr­(VI) ions and the adsorbent surface with increasing temperature. However, considering the moderate variation of the Temkin parameters, temperature had a limited influence on the adsorption mechanism, which remained governed mainly by surface interactions between Cr­(VI) species.^46^

The adsorption capacity of Cr­(VI) in CA-H_3_PO_4_ was compared with different adsorbents reported in the literature, as shown in Table 7.

**Table 7:** Comparison of Cr­(VI) Adsorption Capacities of Some Adsorbents with That of CA-H_3_PO_4_

precursor material	activating agent	pH	*q*_e_ (mg g^–1^)	reference
mango seed Sterculia guttata	H_3_PO_4_	2	7.8	^31^
Prosopis juliflora shells	H_2_SO_4_	6	9.5	^12^
apple shells	H_3_PO_4_	2	36.0	^3^
Aegle marmelos shells	ZnCl_2_	2	43.5	^27^
walnut shells	ZnCl_2_	2	46.2	^8^
walnut shells	H_3_PO_4_	2	74.9	^7^
chestnut shells	H_3_PO_4_	2	85.5	^26^
Sterculia guttata shells	ZnCl_2_	2	90.9	^28^
peanut shells	H_3_PO_4_	2	106.4	^11^
jatropha wood	KOH	2	140.8	^11^
apple shells	H_2_SO_4_	1.8	151.5	^9^
longan seeds	NaOH	3	169.5	^26^
tropical almond shell (Terminalia catappa L.*)*	H_3_PO_4_	2	173.7	present study

#### 3.2.4 Adsorption Process Thermodynamics

The 1/T versus ln *K*_C_ data were plotted and presented a good linear correlation (*R* ^2^ = 0.974), from the slope (−159.7) and intercept (5.374) values, the thermodynamic parameters Δ*G*, Δ*S*, and Δ*H* were calculated, which are listed in Table 8. It can be observed that negative values of Δ*G* increase slowly with decreasing temperature, revealing that the adsorption process is spontaneous at temperatures of 298 and 308 K. Positive Δ*S* indicates an increase in the degree of freedom (or disorder) of Cr­(VI) adsorbed on CA-H_3_PO_4_, and that the adsorption process is controlled by entropic contributions rather than enthalpy contributions.^8^ ^,^ ^47^ In addition, the positive Δ*H* value confirms the endothermic nature of the adsorption, the process being favored by the temperature increase.

**Table 8:** Thermodynamic Parameters for Cr (VI) Adsorption in CA-H_3_PO_4_

temperature (K)	Δ*G* (kJ mol^–1^)	Δ*H* (kJ mol^–1^)	Δ*S* (J mol^–1^ K^–1^)
288	0.414	13.250	44.682
298	–0.156		
308	–0.474		

The Δ*G* value for the 288 K temperature is positive, indicating nonspontaneity of the process. The same behavior was observed by Li et al.,^48^ in his study related to Cr­(VI) adsorption in hydroxy-functionalized ionic liquids (ILs) modified with magnetic chitosan/graphene oxide (MG-ILs–OH).

#### 3.2.5 Regeneration Cycles

Activated carbon can be regenerated using NaOH 0.5 mol L^–1^ as a leach solution, ensuring a 95% efficiency in the first cycle, as shown in Figure 9. The Cr­(VI) adsorption capacity was still at 83.6 mg g^–1^, with 55% of the initial capacity after the fourth regeneration cycle. Even losing 45% of efficiency, the adsorbent remains with expressive adsorption capacity when compared to other studies reported in the literature, already presented in Table 7. This regenerative property of CA-H_3_PO_4_ attributes a low cost to the adsorption process and can be applied to remove Cr­(VI) for five cycles, maintaining the adsorption capacity above 80 mg g^–1^. Moreover, this performance demonstrates the structural stability of the adsorbent under alkaline regeneration conditions, highlighting its potential for repeated use in practical wastewater-treatment applications.

**Figure 9:**
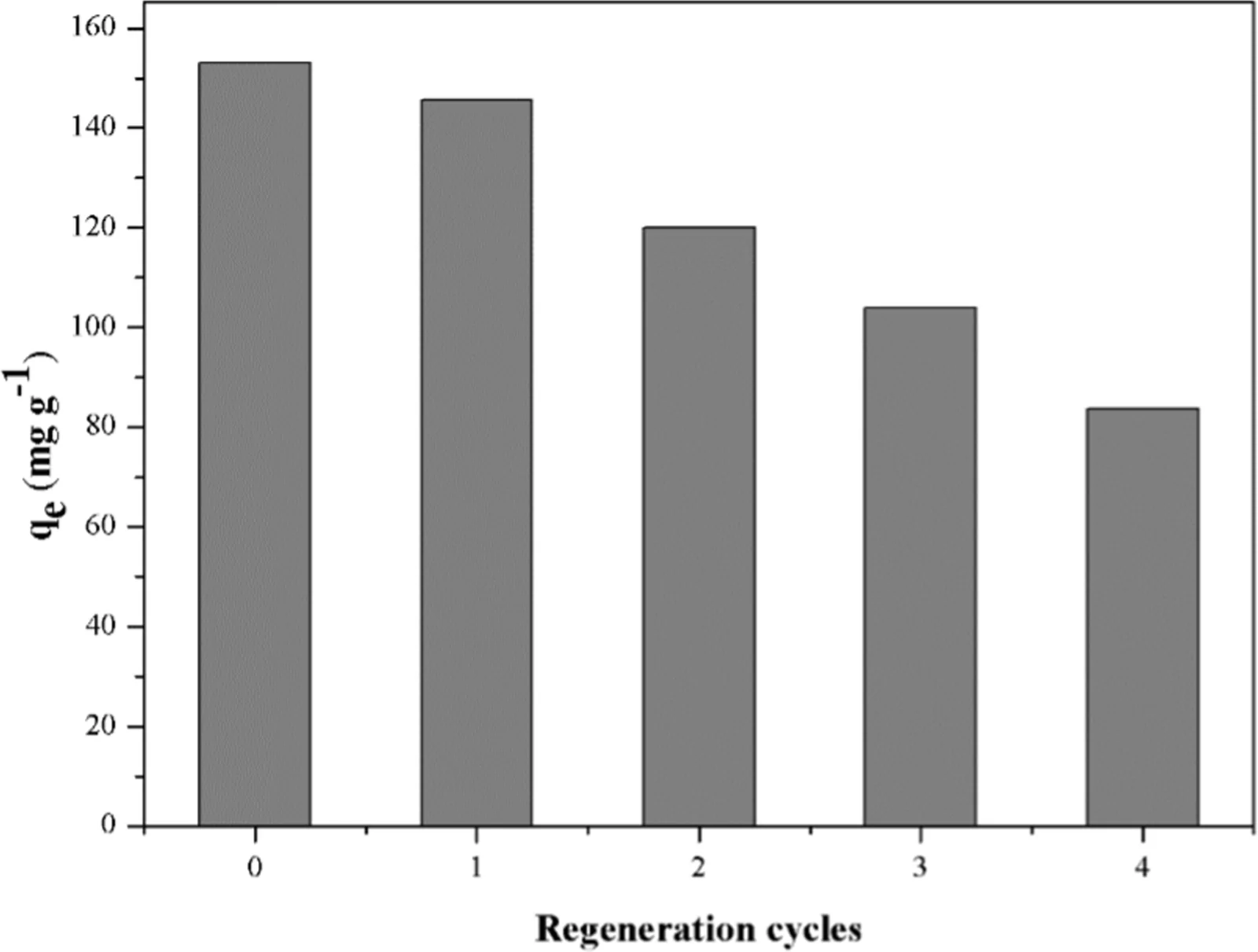
Cr­(VI) adsorption capacity by H_3_PO_4_ after successive regeneration cycles.

This result is significant from both technological and environmental perspectives. The persistence of appreciable adsorption capacity after repeated regeneration indicates that the material should not be regarded as a single-use biosorbent. Instead, the results demonstrate a functional adsorption–regeneration–reuse pathway, which can potentially reduce the consumption of fresh adsorbent, decrease the amount of spent adsorbent requiring disposal, and improve the overall resource efficiency of adsorption-based treatment.

## 4 Conclusions

In the present study, micro and mesoporous activated carbon was produced by chemical activation from the tropical almond shell (*Terminalia catappa L.*), resulting in a product with a high-surface area and well-developed pore structure, which was applied in the adsorption of Cr­(VI) in liquid medium and subsequently evaluated in regeneration cycles using an alkaline leaching solution. The results showed that the prepared adsorbent presented high metal removal efficiency at pH 2. From PCZ analysis, it was observed that the adsorbent surface is protonated in the whole pH range, favoring the adsorption of negatively charged species. Adsorption capacity reached the maximum value of 173.7 mg g^–1^. Adsorption equilibrium was satisfactorily described by the Freundlich isotherm among the Langmuir, Freundlich, and Temkin models evaluated, while the pseudo-second-order model provided the best description of the adsorption kinetics, with the Weber–Morris intraparticle diffusion model indicating that intraparticle diffusion was involved but was not the sole rate-controlling mechanism. The thermodynamic parameters Δ*G*, Δ*S*, and Δ*H* indicated a viable, spontaneous, and endothermic adsorption in the range of 25 to 35 °C. Due to its endothermic nature, the adsorption process is favored with increasing temperature, giving the adsorbent the potential for real effluent applications without prior cooling. Moreover, a particularly relevant finding of this study is that the activated carbon could be regenerated with 0.5 mol L^–1^ NaOH and successfully returned to the adsorption process over successive cycles. Even after the fourth regeneration cycle, the material retained a Cr­(VI) adsorption capacity of 83.6 mg g^–1^, corresponding to 55.0% of its original adsorption capacity. Thus, despite the expected progressive loss of active adsorption sites during repeated regeneration, the adsorbent maintained a substantial absolute adsorption capacity after four cycles.
